# Die Maßnahme Blaufeuer für Erwerbstätige mit psychischer Belastung und gleichzeitiger Arbeitsplatzproblematik – Wird die Zielgruppe erreicht?

**DOI:** 10.1007/s00103-024-03907-4

**Published:** 2024-06-19

**Authors:** Michael Schuler, Christian Gerlich, Lorenz Leven, Silvan Renz, Ina Pamperin, Nadine Vorsatz, Heiner Vogel

**Affiliations:** 1 Institut für Klinische Epidemiologie und Biometrie, Universität Würzburg, Würzburg (Bayern), Deutschland; 2 Department für Angewandte Gesundheitswissenschaften, Hochschule für Gesundheit, Bochum (Nordrhein-Westfalen), Deutschland; 3 Arbeitsgruppe Rehabilitationswissenschaften im Zentrum Psychische Gesundheit, Universitätsklinikum Würzburg, Würzburg (Bayern), Deutschland; 4 Deutsche Rentenversicherung Bund, Berlin, Deutschland

**Keywords:** Versorgung, Psychische Beeinträchtigung, Arbeitsfähigkeit, Beratung, Fallmanagement, Health service, Mental burden, Work ability, Counselling, Case management

## Abstract

**Hintergrund:**

Erwerbstätige mit psychischer Belastung und gleichzeitiger Arbeitsplatzproblematik haben ein erhöhtes Risiko für Chronifizierungen und vorzeitige Frühberentung aufgrund von Erwerbsminderung. Zur Unterstützung dieser Personengruppe wurde im Rahmen des Bundesprogramms rehapro das Projekt „SEMpsych“ (Systemisches Eingliederungsmanagement bei Menschen mit psychischen Beeinträchtigungen) von der Deutschen Rentenversicherung Bund entwickelt. Das Beratungsangebot „Blaufeuer“ wurde in 3 Modellregionen (Berlin, Köln, Nürnberg) implementiert und umfasst in der Regel bis zu 12 Beratungen in 12 Monaten. In diesem Beitrag werden die Charakteristika der Teilnehmenden beschrieben und es wird geprüft, ob sie zur anvisierten Zielgruppe gehören.

**Methode:**

Im Zeitraum 09/2020–06/2022 füllten die Teilnehmenden zwischen der ersten und zweiten Sitzung einen Fragebogen aus. Erfasst wurden soziodemografische, klinische und arbeitsbezogene Variablen. Die Daten werden durch deskriptive Statistiken und 95 %-Konfidenzintervalle ausgewertet.

**Ergebnisse:**

Daten von *n* = 482 Teilnehmenden (66,4 % weiblich; M_Alter_ = 45,2 (±10,2) Jahre; 64,1 % in Vollzeit tätig; 49,8 % derzeit krankgeschrieben) wurden einbezogen. Die Teilnehmenden weisen hohe psychische Beeinträchtigen (z. B. PHQ-9: M = 14,6 (±5,4)) und eine geringe subjektive Arbeitsfähigkeit (z. B. WAS: M = 3,2 (±2,6)) auf. Die meisten berichten von Überforderung auf der Arbeit und geben Probleme mit Vorgesetzten an.

**Diskussion:**

Die Teilnehmenden weisen psychische Belastungswerte auf, die denen von Patient:innen am Beginn einer ambulanten Psychotherapie bzw. beim Erstkontakt in einer stationären Psychiatrie entsprechen. Mit Blaufeuer wird eine hoch belastete Personengruppe angesprochen, die bislang keine adäquate Behandlung erhält. Weitere Studien zur Prozess- und Ergebnisevaluation stehen an.

## Abstract (en)

**Background:**

Employees with mental burden and simultaneous workplace problems have an increased risk of chronification and disability pension. To support this group of people, the German Pension Insurance (Deutsche Rentenversicherung Bund) developed the Systemic Integration Management for People with Mental Impairments (SEMpsych) project as part of the rehapro federal program. The Blaufeuer counselling service was implemented in three model regions (Berlin, Cologne, and Nuremberg). The measure usually comprises up to 12 counselling sessions in 12 months. This article describes the characteristics of the participants and examines whether they belong to the intended target group.

**Method:**

During September 2020 and June 2022, the participants completed a questionnaire between the first and second counselling sessions. Socio-demographic-, clinical- and work-related variables were assessed. The data were analysed using descriptive statistics and 95% confidence intervals.

**Results:**

Data from *n* = 482 participants (66.4% female; M_Age_ = 45.2 years (±10.2 years); 64.1% working full-time; 49.8% currently on sick leave) were included. The participants have high psychological impairments (e.g. PHQ-9: M = 14.6 (±5.4)) and low subjective work ability (e.g. WAS: M = 3.2 (±2.6)). Most participants report overload at work and problems with superiors.

**Discussion:**

Participants exhibit mental burden values that correspond to those of patients at the beginning of outpatient psychotherapy or the first contact in an inpatient psychiatric clinic. Blaufeuer addresses a highly stressed group of people who have not yet received adequate treatment. Further studies on process and outcome evaluation will follow.

## Hintergrund

Schätzungen zur Prävalenz psychischer Erkrankungen zeigen, dass in Deutschland ca. ein Drittel der erwachsenen Bevölkerung innerhalb eines Jahres die Diagnosekriterien für mindestens eine psychische Erkrankung erfüllt [1]. Seit Jahren wird ein Anstieg in psychiatrischen Diagnosen dokumentiert [2] und es gibt Hinweise darauf, dass sich durch die COVID-19-Pandemie auch die Prävalenz psychischer Erkrankungen erhöht hat [3]. Psychische Erkrankungen sind die häufigste Ursache für Erwerbsminderungsrenten [4] und stehen an zweiter Stelle bei den Ursachen für Arbeitsunfähigkeitstage (Krankschreibungen; [5]). Wegen der zumeist langen Ausfallzeiten entstehen dadurch hohe indirekte Kosten für die Gesellschaft [6].

Sind psychische Erkrankungen zusätzlich mit Arbeitsplatzproblemen assoziiert, kann ein komplexes Bedingungsgefüge entstehen, das sowohl die psychische Symptomatik als auch die Arbeitsplatzproblematik verschlechtern kann [7]. Beispielsweise gelten überhöhte Arbeitsanforderungen, geringe Kontrolle über Arbeitsprozesse und -ergebnisse, geringe soziale Unterstützung und Mobbingerfahrungen auf der Arbeit als Risikofaktoren für die Entwicklung von psychischen Erkrankungen. Interventionen für diese Personengruppe sollten daher sowohl die psychische Symptomatik als auch die Arbeitsplatzprobleme adressieren.

Grundsätzlich stehen in Deutschland zahlreiche Beratungs- und Interventionsangebote zur Verfügung, sowohl zur Behandlung von psychischer Symptomatik (z. B. ambulante Psychotherapie) als auch zur Bearbeitung arbeitsplatzspezifischer Problematiken (z. B. betriebliches Gesundheitsmanagement, berufliche Reha). Die Betroffenen sind dabei dennoch mit mehreren Schwierigkeiten konfrontiert:

Zum Ersten sind viele der Angebote nur mangelhaft vernetzt [8]. Beispielsweise ist das der Fall in der Regelversorgung von Menschen mit psychischen Erkrankungen, die sich in 3 Bereiche einordnen lässt: die stationäre und ambulante Akutversorgung, die Rehabilitation sowie das betriebliche Eingliederungsmanagement. Die Bereiche arbeiten leider noch immer oft unabhängig voneinander. Regelhafte strukturierte Zuweisungen untereinander oder weitere Formen der Vernetzung sind nur ansatzweise implementiert.

Zum Zweiten stellt die Unübersichtlichkeit des komplexen Versorgungsystems eine Schwierigkeit für Betroffene dar. Schaeffer et al. zeigten, dass bei mehr als 80 % der Deutschen die sogenannte navigationale Gesundheitskompetenz gering ausgeprägt ist [9]. Darunter wird das Wissen, die Motivation und die Fähigkeit eines Menschen verstanden, sich im Hinblick auf die bestmögliche Versorgung für sich selbst und seine Angehörigen im Gesundheitssystem und dessen Organisation und Diensten zurechtzufinden. Große Schwierigkeiten gibt es insbesondere dabei, herauszufinden, welche Rechte man als Patient:in hat und welche Unterstützungsmöglichkeiten es überhaupt gibt. Das zeigt sich empirisch unter anderem darin, dass Rehabilitationsmaßnahmen häufig nur unzureichend oder zu spät in Anspruch genommen werden. Trotz des Grundsatzes „Reha vor Rente“ haben beispielsweise 30–40 % derjenigen, denen eine Erwerbsminderungsrente genehmigt wurde, in den letzten 5 Jahren vor Renteneinritt keine Teilhabe- oder Rehaleistung erhalten [10].

Und zum Dritten ist der Zugang zu den betrieblichen und außerbetrieblichen Angeboten von der Bekanntheit bei den Betroffenen und deren Verfügbarkeit abhängig. Nicht alle Angebote sind überall verfügbar und allen Betroffenen bekannt. Darüber hinaus sind manche Angebote mit sehr langen Wartezeiten verbunden, was zu einer Verschlechterung der Symptomatik führen kann [11].

Frühzeitig organisierte arbeitsplatzorientierte Interventionen können die Erwerbsfähigkeit (z. B. Anzahl der Personen, die nach Krankschreibung an den Arbeitsplatz zurückkehren, oder die Dauer bis zur Rückkehr an den Arbeitsplatz) jedoch positiv beeinflussen [12, 13]. Wenn klinische und arbeitsplatzbezogene Maßnahmen kombiniert werden, könnten diese besonders erfolgversprechend sein [14].

Das Projekt „SEMpsych“ (Systemisches Eingliederungsmanagement bei Menschen mit psychischen Beeinträchtigungen), das von der Deutschen Rentenversicherung Bund in Kooperation mit der Universität und dem Universitätsklinikum Würzburg, der Medical School Berlin (MSB), dem Institut für Qualitätssicherung und Prävention an der Deutschen Sporthochschule Köln (IQPR) sowie den regionalen Berufsförderungswerken Berlin, Köln und Nürnberg durchgeführt und im Rahmen des Bundesprogramms rehapro (§ 11 SGB IX) gefördert wird, hat zum Ziel, Menschen mit psychischen Belastungen und gleichzeitigen beruflichen bzw. arbeitsplatzbezogenen Problemen möglichst niederschwellig und frühzeitig zu unterstützen. Dazu wurde das Beratungsangebot Blaufeuer entwickelt und im Laufe des Jahres 2020 in 3 Modellregionen (Berlin, Köln, Nürnberg) mit Unterstützung der regionalen Berufsförderungswerke implementiert.

Der Name „Blaufeuer“ bezeichnet in der Schifffahrt ein Leuchtsignal, das in Not geratene Schiffe abgeben können, um eine:n ortskundige:n Lots:in an Bord zu rufen, der/die bei der Navigation in sichere Gewässer unterstützt. In vergleichbarer Weise helfen die Blaufeuerberater:innen (oder: Blaufeuerlots:innen) den Klient:innen sowohl bei der Navigation durch das Gesundheitssystem als auch bei der Sortierung der täglichen Probleme (Daily Hassles, [15]) und der Formulierung der Behandlungswünsche und -ziele. In die Beratungsleistung sind Elemente des Fallmanagements [16] integriert.

Die Zielgruppe von Blaufeuer umfasst zum Zeitpunkt der Kontaktaufnahme Erwerbstätige im Alter von 18 bis 64 Jahren, die sich selbst als „psychisch belastet“ beschreiben und bei denen nach Selbstauskunft gleichzeitig eine damit assoziierte Arbeitsplatzproblematik vorliegt. Das Vorliegen einer ärztlich oder psychotherapeutisch gestellten Diagnose einer psychischen Erkrankung ist nicht notwendig für die Teilnahme an Blaufeuer (die Begriffe „psychisch belastet“ oder „psychische Belastung“ werden hier sowie im ganzen Manuskript nicht im Sinne der DIN EN ISO 10075‑1 als von außen auf den Menschen einwirkende Einflüsse verwendet, sondern im umgangssprachlichen Gebrauch als Synonym für „psychisch unwohl“, „gestresst“, „beansprucht“ usw. herangezogen). Ausschlusskriterien sind eine fehlende positive Prognose für den Verbleib im bzw. die Rückkehr an den Arbeitsplatz innerhalb des Beratungszeitraums (12 Monate), Beratungsunfähigkeit (z. B. durch eine nichtbehandelte schwerwiegende psychiatrische Erkrankung wie akute schizophrene Phasen) und die Teilnahme an einer beruflichen Umschulung, die den Beratungszeitraum überschreitet.

Das Angebot umfasst in der Regel bis zu 12 Beratungssitzungen in einem Zeitrahmen von maximal 12 Monaten (mit der Möglichkeit der Verlängerung). Auf Basis einer umfänglichen Problemanamnese wird in Zusammenarbeit mit den Klient:innen ein individueller Ziel- und Handlungsplan (ZHP) mit entsprechenden Lösungsstrategien erstellt. Ein zentraler Baustein des ZHPs sind für die individuelle Problematik der Klient:innen passende Angebote des Versorgungsystems. Die Berater:innen vermitteln in die Angebote, stimmen ggf. verschiedene Ansätze aufeinander ab bzw. vernetzen diese miteinander. Zur bedarfsgerechten Vermittlung wurde an jedem Standort ein Netzwerk aus bestehenden Angeboten des Versorgungssystems aufgebaut. Um Blaufeuer für potenziell Interessierte bekannter zu machen, sind an den Standorten Informationsveranstaltungen mit Ärzt:innen und Psychotherapeut:innen sowie Betrieben und weiteren Netzwerkpartnern durchgeführt worden. Zusätzlich werden Versicherte von kooperierenden Krankenkassen im Rahmen des Krankengeldmanagements auf das Angebot aufmerksam gemacht [17].

Das Gesamtprojekt wird von 11/2019–04/2025 durchgeführt und ist in 4 Phasen aufgeteilt: die Entwicklungs‑, Implementierungs‑, Bewährungs- und Verstetigungsphase. Die wissenschaftliche Evaluation umfasst eine formative Evaluation, die die Entwicklungs- und Implementierungsphase einschließt, sowie eine sich anschließende summative Evaluation. In der formativen Evaluation werden Fragen des Zugangs (Wer nimmt teil?), des Prozesses (Was geschieht in der Maßnahme?) und der Ergebnisse (Was verändert sich?) untersucht. Die summative Evaluation prüft, inwiefern Veränderungen kausal auf die Maßnahme zurückgeführt werden können.

In diesem Beitrag werden Ergebnisse zur Zugangsevaluation vorgestellt. Wie beschrieben genügt für die Teilnahme eine Selbstbeschreibung als „psychisch belastet“ und dass diese Belastung mit einer Arbeitsplatzproblematik assoziiert ist. Es wird folglich die Frage untersucht, in welchem Ausmaß sich bei einer mit diesen Zugangskriterien erreichten Personengruppe eine psychische Belastung und eine Arbeitsplatzproblematik bei Verwendung von validierten standardisierten Messinstrumenten aufzeigen lässt.

## Methode

### Studiendesign

Alle Teilnehmenden von Blaufeuer wurden gebeten, zwischen der ersten und zweiten Sitzung (T0) sowie nach 3/6/12/18 Monaten einen standardisierten Fragebogen auszufüllen. In die Analysen wurden alle Teilnehmenden eingeschlossen, die zwischen dem 01.09.2020 und dem 30.06.2022 eine Blaufeuerberatung erhielten und zu T0 einen Fragebogen ausgefüllt hatten (*N* = 482).

### Messinstrumente

Neben soziodemografischen (z. B. Alter, Geschlecht) und sozialmedizinischen (z. B. Arbeitsunfähigkeitszeiten) Basisdaten wurden folgende Variablen erfasst:

#### Psychische Gesundheit

##### *Depressive Symptomatik (PHQ-9)*.

Die depressive Symptomatik wurde mit dem Fragebogen PHQ‑9 erhoben [18]. Dieser erfasst mit 9 Items (4-stufige Antwortskala, von *überhaupt nicht* bis *beinahe jeden Tag*) die Kernsymptomatik depressiver Störungen. Die Summenskala kann Werte zwischen 0 und 27 annehmen, wobei höhere Werte eine stärkere Symptomatik andeuten. Werte ≥ 10 werden als klinisch auffällig interpretiert.

##### *Angstsymptomatik (GAD-2)*.

Die Angstsymptomatik wurde mittels GAD‑2 erfasst [19]. Dieser Kurzfragebogen besteht aus 2 Items und die Antwortskala entspricht der des PHQ‑9. Der Skalenwert reicht von 0 bis 6; höhere Werte zeigten eine höhere Symptomatik an. Werte ≥ 3 werden als klinisch auffällig interpretiert.

##### *Somatische Symptome (SSS-8)*.

Die Somatic Symptom Scale (SSS-8) erfasst mittels 8 Items (5-stufige Antwortskala von *gar nicht* bis *sehr stark*) typische, aber medizinisch unspezifische somatische Symptome [20]. Der summierte Skalenwert reicht von 0 bis 32. Höhere Werte zeigen eine höhere Belastung an.

##### *Gesundheitsbezogene Lebensqualität (SF-12)*.

Die körperliche und psychische gesundheitsbezogene Lebensqualität wurde mit dem SF-12 V1.0 erhoben [21]. Werte von 50 gelten als normal. Bei einer Abweichung von mehr als 9 Punktwerten wird von relevanter Einschränkung der Lebensqualität ausgegangen.

##### *Burnout (MBI)*.

Burnout wurde mit dem Maslach Burnout Inventory (MBI) erfasst [22]. Der MBI besteht aus 22 Items (7-stufige Antwortskala von *nie* bis *jeden Tag*), die den 3 Skalen berufliche Erschöpfung (Wertebereich 0–54), Depersonalisation (Wertebereich 0–30) sowie eigene Leistungseinschätzung (Wertebereich 0–48) zugeordnet werden. Höhere Werte in beruflicher Erschöpfung und Depersonalisation zeigen eine höhere Belastung, während höhere Werte in der eigenen Leistungseinschätzung geringere Belastung anzeigen.

#### Arbeitsfähigkeit

*Die subjektive Arbeitsfähigkeit (Work Ability [WA])* wurde mit einer verkürzten 4‑Item-Version [23] des Work Ability Index (WAI) erfasst [24]. Ein Item erfasst die allgemeine Einschätzung der aktuellen Arbeitsfähigkeit in Relation zur besten je erreichten Arbeitsfähigkeit (Work Ability Score [WAS]), die 3 weiteren erfassen die WA im Hinblick auf physische, psychische und soziale Arbeitsanforderungen. Das Antwortformat bildet jeweils eine Skala von 0 *völlig arbeitsunfähig* bis 10 *beste je erreichte Arbeitsfähigkeit*.

Die Antwortwerte der 4 Items werden als Index gemittelt, das WAS-Item wird zusätzlich einzeln ausgewertet.

##### *Subjektive Erwerbsprognose (SPE)*.

Die SPE-Skala [25] setzt sich aus 3 Items zusammen, die zu einem Index zusammengefasst werden. Der Index kann Werte zwischen 0 und 3 annehmen wobei höhere Werte eine höhere Gefährdung der Teilhabe am Erwerbsleben anzeigen. Punktwerte ab 2 können als Marker für eine erhöhte Gefährdung der längerfristigen Teilhabe am Erwerbsleben angesehen werden.

#### Arbeitsbezogene Anforderungen und Ressourcen

##### *Problembereiche am Arbeitsplatz* (Eigenkonstruktion).

Mittels 10 Einzelfragen wurden charakteristische Problembereiche und fehlende Ressourcen in Beruf und Arbeit erhoben: Überforderung, Unterforderung, Arbeitsplatzunsicherheit, fehlende Identifikation mit der Arbeitsstelle, fehlender Handlungsspielraum, Unzufriedenheit mit Rahmenbedingungen, fehlende Weiterbildungs- und Aufstiegsmöglichkeiten, Konflikte oder Unzufriedenheit mit Kolleg:innen und mit Vorgesetzten sowie Ausgrenzung, Mobbing oder sexuelle Belästigung. Für jeden Problembereich konnte auf einer 5‑stufigen Skala angegeben werden, wie stark dieser auf die eigene Situation zutrifft. Höhere Einzelwerte zeigen das höhere Zutreffen der Problematik an. Für die beschreibende Darstellung wurden 2 der zustimmenden Antwortoptionen (*trifft eher zu – trifft stark zu*) zusammengefasst.

##### Arbeitszufriedenheit (KAFA).

Der KAFA [26] untergliedert sich in 6 Teilskalen (Zufriedenheit mit der Tätigkeit, mit Arbeitskolleg:innen, mit Entwicklungsmöglichkeiten, mit der Bezahlung und mit Vorgesetzten sowie Arbeitszufriedenheit), die sich jeweils aus 5 Items zusammensetzen, mit je 5‑stufiger Antwortskala, die die Zustimmung zur jeweiligen Aussage abbildet.

##### *Erholungserfahrungen (REQ)*.

Erholungserfahrungen werden mit dem Recovery Experience Questionnaire (REQ) erhoben [27]. Mit dem Verfahren werden 4 Facetten der Erholung im Sinne von Abschalten von der Arbeit, Entspannung (z. B. die Seele baumeln lassen), Mastery (z. B. in der Freizeit Neues zu erlernen) und Kontrolle (z. B. Dinge zu tun, die man will und selbst bestimmt) erfasst. Jeder Bereich wird mit 4 Items erfasst, für die jeweils auf einer 5‑stufigen Antwortskala die Zustimmung zum jeweiligen Aussageninhalt bestimmt wird. Höhere Skalenwerte zeigen eine höhere Erholungserfahrung an.

### Statistische Analysen

Es werden jeweils univariate deskriptive Statistiken wie Mittelwerte, Standardabweichungen und (prozentuale) Häufigkeiten berechnet, teilweise ergänzt um 95 %-Konfidenzintervalle. Fehlende Werte in den verwendeten Skalen/Items liegen zwischen 0,1 % und 8 %. Auf eine Ersetzung durch multiple Imputation o. Ä. wurde verzichtet, stattdessen wurden in die jeweiligen Analysen alle Personen einbezogen, für die in den jeweils verwendeten Variablen vollständige Daten vorlagen (Missing Pairwise). Alle Analysen wurden für die Gesamtstichprobe sowie für die einzelnen Standorte durchgeführt.

## Ergebnisse

Im Folgenden werden die Ergebnisse für die Gesamtstichprobe dargestellt. Vergleichende Analysen haben gezeigt, dass sich die Ergebnisse nicht substanziell zwischen den 3 Standorten Berlin, Köln und Nürnberg unterscheiden, daher wird – außer bei den soziodemografischen und sozialmedizinischen Merkmalen – aus Platzgründen auf die Darstellung der standortspezifischen Ergebnisse verzichtet.

### Soziodemografische und sozialmedizinische Merkmale

Es wurden Daten von 482 Teilnehmenden in die Analysen eingeschlossen. Etwa 2/3 waren weiblich, das mittlere Alter lag bei M = 45,4 Jahren (±10,2). Etwa die Hälfte lebte mit einem festen Partner zusammen. Jeweils knapp 40 % hatten eine Lehre abgeschlossen bzw. besaßen einen Hochschulabschluss. Der größte Teil arbeitete im Angestelltenverhältnis (85,7 %) und ganztags (64,1 %). Die Hälfte der Teilnehmenden war bei Beratungsbeginn krankgeschrieben. Die Daten sind zwischen den Standorten weitgehend vergleichbar, aber in Nürnberg ist der Anteil an weiblichen Teilnehmer:innen geringer als in Berlin und Köln (Tab. 1).

**Tab. 1:** Soziodemografische und sozialmedizinische Merkmale, aufgeteilt nach Standort

Merkmal	Gesamt	Berlin	Köln	Nürnberg
*Weiblich, n (%)*	320 (66,4)	119 (76,3)	95 (70,9)	106 (55,2)
*Alter, M (SD)*	45,5 (10,2)	44,9 (9,4)	45,4 (10,8)	46,1 (10,3)
*Familienstand, n (%)*				
Ledig	239 (49,7)	99 (63,9)	67 (50,0)	73 (38,0)
Verheiratet	152 (31,6)	32 (20,6)	40 (29,9)	80 (41,7)
Geschieden	81 (16,8)	22 (14,2)	25 (18,7)	34 (17,7)
Verwitwet	9 (1,9)	2 (1,3)	2 (1,5)	5 (2,6)
*Ohne festen Partner lebend, n (%)*	251 (52,6)	91 (60,3)	69 (51,5)	91 (47,4)
*Ausbildung, n (%)*				
Keine	18 (3,8)	3 (1,9)	6 (4,6)	9 (4,8)
Lehre	185 (39,1)	50 (32,1)	52 (40,0)	83 (43,9)
Fachschule/Meister	72 (15,2)	20 (12,8)	12 (9,2)	40 (20,8)
Hochschule (FH/Uni)	196 (41,4)	80 (51,9)	59 (45,4)	57 (30,2)
Andere	2 (0,4)	1 (0,6)	1 (0,8)	0
*Beruflicher Status, n (%)*				
Arbeiter:in	35 (7,3)	11 (7,1)	7 (5,3)	17 (8,9)
Angestellt	413 (85,7)	131 (84,0)	120 (90,2)	162 (84,4)
Verbeamtet	22 (4,6)	8 (5,1)	4 (3,0)	10 (5,2)
Selbstständig	9 (1,9)	6 (3,8)	2 (1,5)	1 (0,5)
Sonstiges	2 (0,4)	0	0	2 (1,0)
*Erwerbstätigkeit, n (%)*				
Ganztags	303 (64,1)	79 (52,3)	88 (66,7)	136 (71,6)
Halbtags	138 (29,2)	57 (37,7)	38 (28,8)	43 (22,6)
< halbtags	6 (1,2)	3 (2,0)	1 (0,8)	2 (1,1)
In Aus‑/Fort‑/Weiterbildung	7 (1,2)	3 (2,0)	3 (2,2)	1 (0,5)
Sonstiges	19 (3,9)	9 (6,0)	2 (1,5)	8 (4,2)
*Haushaltseinkommen, n (%)*				
< 1500 €	58 (12,3)	25 (26,3)	14 (10,9)	19 (10,1)
1500–3000 €	254 (54,0)	85 (55,6)	72 (55,8)	97 (51,6)
> 3000 €	158 (33,6)	43 (28,1)	43 (33,3)	72 (38,3)
*Schwerbehindertenausweis, n (%)*				
Nein	372 (78,6)	119 (78,8)	101 (76,5)	152 (80,0)
Ja	69 (14,6)	20 (13,8)	18 (9,8)	31 (16,3)
Beantragt	32 (6,8)	12 (7,9)	13 (13,6)	7 (3,7)
*Aktuell krankgeschrieben (AU), n (%)*	240 (49,8)	76 (49,7)	62 (46,6)	98 (51,6)
*Dauer aktuelle AU*^a^*, n (%)*				
Bis zu 1 Woche	8 (3,7)	4 (6,2)	1 (1,5)	3 (3,6)
2–6 Wochen	41 (19,1)	15 (23,1)	13 (19,7)	13 (15,5)
> 6 Wochen–3 Monate	52 (24,2)	19 (29,2)	13 (19,7)	20 (23,8)
> 3 Monate–6 Monate	50 (23,3)	15 (23,1)	18 (27,3)	17 (20,2)
> 6 Monate–1 Jahr	35 (16,3)	6 (9,2)	14 (21,2)	15 (17,9)
> 1 Jahr	29 (13,5)	6 (9,2)	7 (10,6)	16 (19,0)
*AU letzte 12 Monate, n (%)*				
Bis zu 1 Woche	36 (18,9)	10 (16,4)	9 (19,1)	17 (20,7)
2–6 Wochen	107 (56,3)	32 (52,5)	30 (63,8)	45 (54,9)
> 6 Wochen–3 Monate	20 (10,5)	5 (8,2)	4 (8,5)	11 (13,4)
> 3 Monate–6 Monate	12 (6,3)	5 (8,2)	1 (2,1)	6 (7,3)
> 6 Monate	15 (7,9)	9 (14,8)	3 (6,4)	3 (3,7)

*AU* Arbeitsunfähigkeit; *M* Mittelwert; *SD* Standardabweichung

^a^Von 240 Teilnehmenden, die aktuell krankgeschrieben sind, liegen von *n* = 215 Angaben zur AU-Dauer vor

### Psychische Belastung

Die Teilnehmenden weisen zu Beratungsbeginn bei fast allen Indikatoren der psychischen Belastung hohe Werte auf (Tab. 2). Die mittlere Belastung im PHQ‑9 liegt mit M = 14,6 (±5.4) im hohen Bereich. Auch im GAD‑2 und SSS‑8 finden sich hohe Mittelwerte. Im SF-12 liegt der Mittelwert in der körperlichen Lebensqualität (M = 45,1 (±10,1)) deutlich über dem der psychischen Lebensqualität (M = 28,3 (±8,3)), was eine starke psychische, aber geringe körperliche Einschränkung anzeigt. Im MBI zeigen die Werte in der Subskala „berufliche Erschöpfung“ (M = 35,5 (±10,4)) ebenfalls eine hohe Beeinträchtigung an.

**Tab. 2:** Psychische Belastung und Arbeitsbelastung

Instrument	*n*	M [95 %-KI]	SD
PHQ‑9	467	14,6 [14,1; 15,1]	5,4
GAD‑2	477	3,9 [3,8; 4,1]	1,63
SSS‑8	452	13,9 [13,3; 14,5]	6,06
SF-12 körperlich	439	45,1 [44,2; 46,0]	10,1
SF-12 psychisch	439	28,3 [27,5; 29,1]	8,3
MBI – berufliche Erschöpfung	447	35,5 [34,5; 36,5]	10,4
MBI – Depersonalisation	439	9,9 [9,3; 10,5]	6,9
MBI – Leistungseinschätzung	422	23,0 [22,2; 23,8]	8,5
WA	468	3,8 [3,6; 4,0]	2,2
WAS	473	3,2 [3,0; 3,4]	2,6
SPE	444	1,3 [1,2; 1,4]	1,0
REQ – Abschalten	475	2,3 [2,2; 2,4]	0,9
REQ – Entspannung	468	2,8 [2,7; 2,9]	0,7
REQ – Mastery	474	2,7 [2,6; 2,8]	0,9
REQ – Kontrolle	471	3,5 [3,4; 3,6]	0,9

*n* Anzahl, M Mittelwert, *SD* Standardabweichung, *KI* Konfidenzintervall, *PHQ‑9* Patient Health Questionnaire – 9, *GAD‑2* General Anxiety Disorder Questionnaire – 2, *SSS‑8* Somatic Symptom Scale – 8, *SF-12*: Short Form-12, *MBI* Maslach Burnout Inventory, *WA* Work Ability, *WAS* Work Ability Score, *SPE* Skala subjektive Erwerbsprognose, *REQ* Erholungsfragebogen/Recovery Questionnaire

### Arbeitsfähigkeit und Arbeitsbelastung

Die subjektive Arbeitsfähigkeit wird sowohl nach WA als auch nach WAS als sehr niedrig eingeschätzt, die Mittelwerte liegen im unteren Bereich der Skala. Der Mittelwert in der SPE-Skala liegt ebenfalls im geringen Bereich. Nimmt man den Cut-off-Wert von 2 Punkten, so zeigt sich bei *N* = 205 (46,2 %) ein erhöhtes Risiko für eine substanziell verminderte Teilhabe am Erwerbsleben. Im Erholungsfragebogen weisen die Teilnehmer:innen vor allem in der Skala „Abschalten“ besonders geringe Werte auf.

Im KAFA zeigt sich mit M = 3,1 (±0,94) eine mittlere allgemeine Arbeitszufriedenheit (Tab. 3). Am geringsten ist sie in den Bereichen „Entwicklungsmöglichkeiten“ und „Vorgesetzte“ ausgeprägt. Im Fragebogen zu den Arbeitsplatzproblemen (AP) geben die meisten Teilnehmer:innen vor allem Überforderung und Probleme mit den Vorgesetzten an. In den anderen Problembereichen (z. B. Konflikte/Unzufriedenheiten mit Kolleg:innen oder Erfahrung mit Ausgrenzung, Mobbing, Belästigung) findet sich tendenziell eine Gleichverteilung der Werte. Es gibt demnach einen substanziellen Anteil an Teilnehmenden, die Schwierigkeiten in diesen Bereichen aufweisen, und einen vergleichbaren Anteil ohne Schwierigkeiten in diesen Bereichen (Tab. 4).

**Tab. 3:** Arbeitszufriedenheit und Probleme am Arbeitsplatz

	*n*	M [95 %-KI]	SD
KAFA – Arbeitszufriedenheit	449	3,1 [3,0; 3,2]	0,94
KAFA – Tätigkeit	460	3,5 [3,4; 3,6]	0,9
KAFA – Kollegen	464	3,4 [3,3; 3,5]	0,9
KAFA – Entwicklungsmöglichkeiten	449	2,4 [2,3; 2,5]	1,03
KAFA – Bezahlung	457	3,5 [3,4; 3,6]	1,2
KAFA – Vorgesetzte	459	3,0 [2,9; 3,1]	1,1
AP – Überforderung	480	3,9 [3,8; 4,0]	1,2
AP – Unterforderung	478	2,1 [2,0; 2,2]	1,3
AP – Arbeitsplatzunsicherheit	474	2,4 [2,3; 2,5]	1,5
AP – fehlender Handlungsspielraum	477	3,3 [3,2; 3,4]	1,2
AP – fehlende Identifikation	477	3,0 [2,9; 3,1]	1,2
AP – fehlende Weiterbildungs‑/Aufstiegsmöglichkeiten	478	3,1 [3,0; 3,2]	1,4
AP – Unzufriedenheit mit Rahmenbedingungen	478	3,1 [3,0; 3,2]	1,4
AP – Konflikte/Unzufriedenheit mit Kolleg:innen	479	3,2 [3,1; 3,3]	1,3
AP – Konflikte/Unzufriedenheit mit Vorgesetzten	479	3,8 [3,7; 3,9]	1,3
AP – Ausgrenzung, Mobbing, Belästigung	476	2,4 [2,3; 2,5]	1,6

*N* Anzahl, *M* Mittelwert, *SD* Standardabweichung, *KI* Konfidenzintervall, *KAFA* Kurzfragebogen zur Erfassung von allgemeiner und facettenspezifischer Arbeitszufriedenheit, *AP* Arbeitsplatzprobleme

**Tab. 4:** Probleme am Arbeitsplatz (Prozente bezogen auf Anzahl Personen mit gültigen Werten pro Item, siehe jeweils Tab. 3)

Arbeitsplatzprobleme	Trifft nicht zu	Trifft eher nicht zu	Trifft etwas zu	Trifft eher zu	Trifft stark zu
Überforderung	32 (6,7 %)	42 (8,8 %)	65 (13,5 %)	12 (26,9 %)	212 (44,2 %)
Unterforderung	234 (49,0 %)	98 (20,5 %)	65 (13,6 %)	47 (9,8 %)	34 (7,1 %)
Arbeitsplatzunsicherheit	184 (38,8 %)	87 (18,4 %)	82 (17,3 %)	53 (11,2 %)	68 (14,3 %)
Fehlende Identifikation	93 (19,5 %)	93 (19,5 %)	105 (22,0 %)	108 (22,6 %)	78 (16,4 %)
Fehlender Handlungsspielraum	42 (8,8 %)	87 (18,2 %)	108 (22,6 %)	148 (31,0 %)	92 (19,3 %)
Unzufriedenheit mit Rahmenbedingungen	76 (15,9 %)	93 (19,5 %)	95 (19,9 %)	115 (24,1 %)	99 (20,7 %)
Fehlende Weiterbildungs- und Aufstiegsmöglichkeiten	81 (16,9 %)	97 (20,3 %)	103 (21,5 %)	96 (20,1 %)	101 (21,1 %)
Konflikte oder Unzufriedenheit mit Kolleg:innen	59 (12,3 %)	96 (20,0 %)	107 (22,3 %)	111 (23,2 %)	106 (22,1 %)
Konflikte oder Unzufriedenheit mit Vorgesetzten	27 (5,6 %)	66 (13,8 %)	76 (15,9 %)	124 (25,9 %)	186 (38,8 %)
Ausgrenzung, Mobbing, sexuelle Belästigung	219 (46,0 %)	57 (12,0 %)	61 (12,8 %)	54 (11,3 %)	85 (17,9 %)

## Diskussion

Blaufeuer wendet sich an Personen, die sich selbst als psychisch belastet bezeichnen und bei denen nach Selbstauskunft eine damit assoziierte Arbeitsplatzproblematik vorliegt. Die Ergebnisse der Studie zeigen, dass die Blaufeuer-Klientel dieser Zielgruppe entspricht. Die Teilnehmenden weisen sowohl hohe Werte in Indikatoren der psychischen Gesundheit als auch eine geringe subjektive Arbeitsfähigkeit auf und fast die Hälfte der Teilnehmenden ist zu Maßnahmenbeginn krankgeschrieben. Im Folgenden werden diese Ergebnisse genauer beleuchtet.

Der Mittelwert im PHQ‑9 in der deutschen Allgemeinbevölkerung liegt bei ca. 2,9 Punkten [28], Werte von ≥ 10 werden als klinisch auffällig betrachtet. Bei den Blaufeuer-Teilnehmenden liegt der Wert mit M = 14,6 deutlich über dieser Grenze und entspricht in etwa den Werten von Patient:innen am Beginn einer ambulanten Psychotherapie [29] bzw. bei Erstkontakt einer psychiatrischen Einrichtung [30]. Vergleichbares zeigt sich für den GAD‑2, in dem die Blaufeuer-Teilnehmenden Werte von M = 3,9 aufwiesen. In der deutschen Allgemeinbevölkerung liegen die Werte je nach Studie zwischen 0,75 und 0,95 Punkten [3, 31, 32]. Krebspatient:innen weisen meist im Mittel Werte zwischen 1,2 und 1,9 auf [33, 34]; in einer Studie mit Suchterkrankten in Indien lagen die Werte bei 3,4 [35]. Diese Vergleiche verdeutlichen, dass die Teilnehmer:innen der Blaufeuer-Beratung nicht nur leichte, sondern erhebliche psychische Belastungen aufweisen, mutmaßlich mit Behandlungsbedarf, aber bislang eben keine adäquate Behandlung in Anspruch genommen hatten. Bemerkenswert ist, dass der Hälfte dieses Personenkreises zu Beratungsbeginn Arbeitsunfähigkeit bescheinigt ist, die zweite Hälfte jedoch trotz dieser Belastung zur Arbeit geht. Mit Blaufeuer werden auch Letztere erreicht. Dieses Ergebnis erinnert an das Konzept des Präsentismus, worunter die „… Anwesenheit am Arbeitsplatz trotz gesundheitlicher oder anderweitiger Beeinträchtigung, die eine Abwesenheit legitimiert hätten“, verstanden wird [36]. Präsentismus ist ebenfalls mit psychischen Beeinträchtigungen wie Depressivität assoziiert [37].

Die beruflichen und sozialmedizinischen Probleme spiegeln sich sowohl in der geringen subjektiven Arbeitsfähigkeit, der hohen AU-Quote und der hohen Anzahl an AU-Tagen als auch den subjektiv genannten Problembereichen wider. Auch im WAS als Maß der subjektiven Arbeitsfähigkeit weisen die Blaufeuer-Teilnehmenden mit M = 3,2 sehr geringe Werte auf. So wiesen beispielsweise in einer Studie von Bethge et al. orthopädische Rehabilitanden in der Standard-Reha im Mittel einen Wert von 5,0, diejenigen einer medizinisch-beruflich orientierten Rehabilitationsmaßnahme im Mittel einen Wert von 4,1 auf [38].

Viele Teilnehmende berichten insbesondere von Überlastungen und Problemen mit Vorgesetzten. Allerdings werden auch die anderen Problembereiche (z. B. Probleme mit Kolleg:innen) von vielen Personen genannt. In den entsprechenden Variablen zeigt sich tendenziell eine Gleichverteilung: Es gibt also zum Beispiel in der Klientel viele Personen, die von erheblichen Problemen mit Kolleg:innen berichten, aber auch viele Personen, die mit Kolleg:innen keine Schwierigkeiten haben. Wie diese Unterschiede zu erklären sind und ob sich beispielsweise unterschiedliche „Typen“ von Blaufeuer-Klient:innen unterscheiden lassen, müssen weitere Analysen zeigen.

Unter den Teilnehmenden finden sich an allen 3 Standorten mehr Frauen als Männer, wenngleich die Geschlechtsunterschiede deutlich differierten (55 % in Nürnberg vs. 76 % in Berlin). Der Beschäftigungsanteil von Frauen ist eigentlich geringer als der von Männern. Dass dennoch mehr Frauen Blaufeuer in Anspruch nehmen, könnte damit zusammenhängen, dass Frauen häufiger von affektiven Störungen und Angsterkrankungen betroffen sind als Männer. Sie erleben psychische Belastung in ausgeprägterer Form, berichten höhere Belastungswerte in den Indikatoren zur psychischen Gesundheit [39] und nehmen Hilfsangebote eher in Anspruch als Männer [40].

Im Vergleich zur erwerbstätigen Allgemeinbevölkerung ist unter den Teilnehmenden der Anteil an Personen mit einer höheren Qualifikation überrepräsentiert und es finden sich sehr wenige Teilnehmende ohne qualifizierte Schul- oder Berufsausbildung. Ebenso finden sich im beruflichen Status mehr Angestellte resp. weniger Arbeiter:innen und Selbständige im Vergleich zur erwerbstätigen Allgemeinbevölkerung. Blaufeuer wird damit von einer Personengruppe in Anspruch genommen, die sich in ähnlicher soziodemografischer Zusammensetzung in der Versorgung von Personen mit psychischen Problemen wiederfindet, wie z. B. in der psychosomatischen Rehabilitation [41]. Möglicherweise sind für die weniger präsenten Personengruppen noch andere und spezifische Ansprache- und Zugangswege notwendig.

### Limitationen

Zu den Limitationen der vorliegenden Studie gehört, dass keine ausführliche psychologische und ärztliche Diagnostik vorliegt. Die Daten beruhen auf Fragebögen, die eher für Screeningzwecke geeignet sind als für Diagnostik. Dies erschwert zum einen den Vergleich mit klinischen Stichproben, z. B. aus Studien zur psychotherapeutischen und psychiatrischen Behandlung. Zum anderen ist unklar, ob beispielsweise die Personen mit hohen Werten im PHQ‑9 auch die Diagnosekriterien für eine depressive Erkrankung erfüllen. Eine Diagnose stellt jedoch keine Voraussetzung für die Blaufeuer-Beratung dar, da Blaufeuer keine Therapiemaßnahme, sondern in erster Linie ein Beratungs- und Vermittlungsangebot darstellt. Es kann zwar nicht ausgeschlossen werden, dass die Teilnehmenden sich in den Fragebögen als „belasteter“ darstellen, als sie „tatsächlich“ sind, doch spricht gegen diese Annahme, dass die Teilnahme an Blaufeuer nicht von den Angaben des Fragebogens abhängt und dies den Teilnehmenden zum Zeitpunkt der Fragebogenbearbeitung – zwischen der ersten und der zweiten Sitzung – bekannt war. Des Weiteren handelt es sich um eine anfallende Stichprobe, also um Personen, die aktiv Blaufeuer aufsuchen. Über Personen, die ebenfalls von einer Blaufeuer-Maßnahme profitieren könnten, aber die Maßnahme nicht aufsuchen, können keine Angaben gemacht werden.

Darüber hinaus ist Blaufeuer bislang nur in Berlin, Köln und Nürnberg implementiert. Die 3 Standorte unterscheiden sich zwar in vielen Aspekten wie Größe und soziodemografischer Zusammensetzung, sind aber dennoch nicht repräsentativ für alle Gebiete in Deutschland. Ob sich die Zusammensetzung der Klientel beispielsweise bei einem Blaufeuer-Beratungsangebot in ländlicheren Gebieten unterscheiden würde, ist unklar. Des Weiteren wurde zwar versucht, mittels weitgehend in der Versorgungsforschung bewährter Instrumente relevante Aspekte der psychischen Gesundheit, der Arbeitsfähigkeit und der Arbeitsplatzproblematiken abzubilden, doch sind einige potenziell relevante Aspekte nicht erfasst worden. Hierzu gehören beispielsweise Arbeitsverhältnisse in der Vergangenheit oder familiäre und soziale Unterstützung.

## Fazit

Die Maßnahme Blaufeuer erreicht Erwerbstätige, die deutlich psychisch belastet sind, deren Arbeitsfähigkeit erheblich beeinträchtigt ist und die dennoch bislang keine adäquate Behandlung in Anspruch nehmen. Blaufeuer versucht eine „Vermittlungslücke“ auszufüllen, damit die Betroffenen die für sie passenden Angebote in Anspruch nehmen können. Inwieweit in Blaufeuer die geplanten Maßnahmen umgesetzt werden und ob Blaufeuer für die Zielgruppe hilfreich ist, wird im weiteren Verlauf des Projekts geprüft werden.
