# Erratum zu: Die Maßnahme Blaufeuer für Erwerbstätige mit psychischer Belastung und gleichzeitiger Arbeitsplatzproblematik – Wird die Zielgruppe erreicht?

**DOI:** 10.1007/s00103-025-04117-2

**Published:** 2025-08-19

**Authors:** Michael Schuler, Christian Gerlich, Lorenz Leven, Silvan Renz, Ina Pamperin, Nadine Vorsatz, Heiner Vogel

**Affiliations:** 1 Institut für Klinische Epidemiologie und Biometrie, Universität Würzburg, Würzburg (Bayern), Deutschland; 2 Department für Angewandte Gesundheitswissenschaften, Hochschule für Gesundheit, Bochum (Nordrhein-Westfalen), Deutschland; 3 Arbeitsgruppe Rehabilitationswissenschaften im Zentrum Psychische Gesundheit, Universitätsklinikum Würzburg, Würzburg (Bayern), Deutschland; 4 Deutsche Rentenversicherung Bund, Berlin, Deutschland

**Erratum zu:**

**Bundesgesundheitsbl 2024**

10.1007/s00103-024-03907-4

Der Artikel „Die Maßnahme Blaufeuer für Erwerbstätige mit psychischer Belastung und gleichzeitiger Arbeitsplatzproblematik – Wird die Zielgruppe erreicht?“, geschrieben von Michael Schuler, Christian Gerlich, Lorenz Leven, Silvan Renz, Ina Pamperin, Nadine Vorsatz und Heiner Vogel, wurde ursprünglich unter exklusiver Lizenz von Springer-Verlag GmbH Deutschland, ein Teil von Springer Nature 2024 veröffentlicht. Als Ergebnis der anschließenden Entscheidung den Artikel im Open-Access-Modell zu veröffentlichen, wurde der Copyright-Vermerk des Artikels am 31 Juli 2025 in © The Author(s) 2025 geändert und der Artikel wird nun unter der Creative Commons Namensnennung Creative Commons Namensnennung 4.0 International Lizenz veröffentlicht, welche die Nutzung, Vervielfältigung, Bearbeitung, Verbreitung und Wiedergabe in jeglichem Medium und Format erlaubt, sofern Sie den/die ursprünglichen Autor(en) und die Quelle ordnungsgemäß nennen, einen Link zur Creative Commons Lizenz beifügen und angeben, ob Änderungen vorgenommen wurden.

Die in diesem Artikel enthaltenen Bilder und sonstiges Drittmaterial unterliegen ebenfalls der genannten Creative Commons Lizenz, sofern sich aus der Abbildungslegende nichts anderes ergibt. Sofern das betreffende Material nicht unter der genannten Creative Commons Lizenz steht und die betreffende Handlung nicht nach gesetzlichen Vorschriften erlaubt ist, ist für die oben aufgeführten Weiterverwendungen des Materials die Einwilligung des jeweiligen Rechteinhabers einzuholen.

Weitere Details zur Lizenz entnehmen Sie bitte der Lizenzinformation auf http://creativecommons.org/licenses/by/4.0/deed.de.

Open Access gefördert durch: Open access funding enabled and organized by Projekt DEAL.

